# Chemical profiles, pharmacological properties, and *in silico* studies provide new insights on *Cycas pectinata*

**DOI:** 10.1016/j.heliyon.2020.e04061

**Published:** 2020-06-04

**Authors:** Abu Montakim Tareq, Saifuddin Farhad, A.B.M. Neshar Uddin, Muminul Hoque, Mst. Samima Nasrin, Mir Md. Rokib Uddin, Mohiminul Hasan, Arafat Sultana, Mst. Shirajum Munira, Chadni Lyzu, S.M. Moazzem Hossen, A.S.M. Ali Reza, Talha Bin Emran

**Affiliations:** a Department of Pharmacy, International Islamic University Chittagong, Kumira, Chittagong 4318, Bangladesh; b Society for Interdisciplinary Research and Innovation, Chawkbazar, 4203, Chittagong, Bangladesh; c Department of Biochemistry and Molecular Biology, University of Chittagong, Chittagong 4331, Bangladesh; d Department of Pharmacy, Southeast University, Dhaka 1212, Bangladesh; e Biomedical and Toxicological Research Institute, Bangladesh Council of Scientific and Industrial Research (BCSIR), Dr. Qudrat-I-Khuda Road, Dhanmondi, Dhaka 1205, Bangladesh; f Department of Pharmacy, Faculty of Biological Science, University of Chittagong, Chittagong 4331, Bangladesh; g Department of Pharmacy, BGC Trust University Bangladesh, Chittagong 4381, Bangladesh

**Keywords:** *Cycas pectinata*, Antioxidant, Anxiolytic, Analgesic, Anti-inflammatory, Plant biology, Bioinformatics, Pharmaceutical science, Alternative medicine, Evidence-based medicine

## Abstract

The current study aimed to qualitatively and quantitatively determine the phytochemical components of *Cycas pectinata* methanol extract (MECP), along with its antioxidant, anti-inflammatory, thrombolytic, locomotor, anxiolytic, analgesic, and antidiarrheal activities. The *in vitro* antioxidant activity was evaluated by DPPH scavenging assay and the total phenol and total flavonoid contents, while the anti-inflammatory activity was evaluated by a protein denaturation assay. The *in vivo* locomotor effects were examined using the open field test and hole-cross test. The anxiolytic effect was examined using the elevated plus maze (EPM) test, hole-board test (HBT), and light–dark test (LDT), while the analgesic activity was investigated using the acetic acid-induced writhing test. The antidiarrheal effect was evaluated by castor oil-induced diarrhea and gastrointestinal motility. Ten bioactive compounds were selected on the basis of their biological activities and further investigated using *in silico* molecular docking simulation to correlate with the identified pharmacological properties. Additionally, the ADME properties of the compounds were evaluated according to their drug-likeness profile. MECP had a maximum total phenol content of 209.85 ± 3.40 gallic acid equivalents/g extract and a total flavonoid content of 105.17 ± 3.45 quercetin equivalents/g extract, with an IC_50_ value of 631.44 μg/mL. MECP (62.5–500 μg/mL) elicited 20.96–38.12% decreased protein denaturation compared to diclofenac sodium (65.40–83.50%), while a 35.72% (P < 0.001) clot lysis activity was observed for the 10 mg/mL concentration. MECP induced a dose-dependent reduction in locomotor activity, with a significant anxiolytic effect. In the analgesic test, MECP (200, 400 mg/kg) showed a 45.12% and 58.82% inhibition in analgesia, and the 400 mg/kg dose elicited a 27.5% inhibition in intestinal motility. These findings suggest that MECP might be effective in treating antioxidant, anti-inflammatory, and neuropharmacological defects, but this requires further study.

## 1 Introduction

The importance of medicinal plants in alleviating various human disorders is well-known (Okwu and Uchenna, 2009). Over 25% of modern remedies or drugs have either directly or indirectly come from plants. The plant kingdom is an immense source of prospective medications, and the use of medicinal plants is continually increasing (Maity et al., 2009). Medicinal plants have been associated with several pharmacological activities such as anxiolytic, antipyretic, cardioprotective, anti-inflammatory immunomodulatory, and antinociceptive activities (Okwu and Uchenna, 2009). Drugs that are sourced from plants are effectively accessible and inexpensive, and they are often safe and have fewer adverse side effects (Uddin et al., 2016).

Reactive oxygen species (ROS) and reactive nitrogen species (RNS) are produced in the human body in the course of normal metabolic processes to complete or achieve normal biological functions. Oxidative stress (OS) is a primary contributor to various diseases, notably including neurological disorders, cancer, ischemic stroke, respiratory disorders, chronic obstructive pulmonary disease (COPD), and atherosclerosis (Reza et al., 2018a). Living cells have an excellent defense mechanism to control free radicals from their damaging effects. This is possible due to the presence of various detoxifying enzymes and metabolites (e.g., superoxide dismutase, peroxidase) in the body that scavenge free radicals. Antioxidants that act indirectly are essential in the defense mechanisms of the body to prevent the damaging effects of ROS/RNS. The human body produces several antioxidants that work to limit the impact of free radical damage. Edible vegetables and dietary fruits are a source of abundant antioxidants (Reza et al., 2018b).

From early civilization, herbal medicines have been effective in the treatment of certain diseases (Hoque et al., 2018). As stated by the World Health Organization (WHO), 80% of the population (mostly in African and Asian countries) relies on herbal remedies in their health care (Ekor, 2014). Nearly 25% of recommended prescriptions contain plant-derived components, and approximately 121 active compounds derived from plants are presently used in pharmaceutical products (Rates, 2001). The assessment of medications is generally founded on phytochemical, pharmacological, and comparable methodologies and includes instrumental procedures such as chromatography, microscopy, and others.

*Cycas pectinata*, locally known as moniraj, belongs to the Cycadaceae family (Islam et al., 2016). It is traditionally used in hair loss (Nair and van Staden, 2012), while local people used it to enhance male sexual potency and for stomach ache and ulcers. However, there are very few studies regarding its phytochemical analysis; however, a phytochemical analysis of its microspores and megaspores revealed the presence of alkaloids, glycosides, flavonoids, terpenoids, and steroids (Bhowmik and Datta, 2014). Understanding the chemical composition and biological activity of plants is vital to evaluate therapeutic substances and to assess new potential agents such as tannins, oils, gums, and precursors that could be used in complex therapeutic agents.

To the best of our knowledge, no previous study has reported the pharmacological activities of *C. pectinata*. For this reason, we investigated its phytochemical composition by gas chromatography and spectrometric measurement (GC-MS), along with an assessment of the *in vitro* antioxidant, anti-inflammatory, thrombolytic, and *in vivo* locomotor, anxiolytic, analgesic, and antidiarrheal activities of the methanol extract of *C. pectinata*.

## 2 Materials and methods

### 2.1 Chemicals

Diazepam, diclofenac sodium, and loperamide (Square Pharmaceuticals Ltd. Dhaka, Bangladesh), acetic acid (Merck, Mumbai, India), castor oil (Sigma Aldrich Co., St. Louis, USA) and all other chemicals were obtained from local trader through Taj Scientific Ltd.

### 2.2 Experimental animals

Male and female Swiss albino mice weighing 20–30 g were obtained from the Department of Pharmacy, International Islamic University Chittagong, Bangladesh, at six to seven weeks old. The animals were allowed to adapt to the housing environment for 7 days. They were housed in conditions of 25 ± 2 °C with a light/dark cycle of 12 h. They were supplied with pelleted food and adequate water. The study was conducted according to the guidelines of the Planning and Development (P&D) Committee of Department of Pharmacy, International Islamic University Chittagong (Pharm/p&d/138/13-’19,22/12/2019).

### 2.3 Collection and preparation of plant extract

Leaves of the *C. pectinata* were collected from the Khagrachari Hill tract area, Chittagong, Bangladesh, in March of 2018 and were authenticated by Md. Anwarul Islam, Department of Botany, Jahangirnagar University, Savar-1342, Bangladesh under accession number Anwar-0941. The plant materials were collected in a fresh condition and were then dried for ten days. The dried material was then ground into a coarse powder using a mechanical grinder. The coarse powder was soaked in methanol for seven days with occasional shaking and stirring. The material was then filtered using Whatman filter paper #1, and the obtained filtrate was evaporated with a water bath to yield a viscous mass. The viscous mass was stored in a refrigerator (4 °C) for future use.

### 2.4 Qualitative phytochemical analysis

The *Cycas pectinata* methanol extract (MECP) was qualitatively screened for bioactive compounds using standard procedures to evaluate the presence of alkaloids, carbohydrates, flavonoids, terpenoids, tannins, saponins, phenols, polyphenol, steroids, cardiac glycosides, glycosides, coumarin, amino acids, resins, quinones, anthraquinones, vitamin C, gums and mucilage, carboxylic acids, phytosterols, and sterols (Evans, 2009; Harborne, 1998; Hossain et al., 2018; Sofowora, 1996).

### 2.5 Quantitative analysis of phytoconstituents

Total phenol and flavonoid was used to evaluate the amount of phytoconstituents, while gas chromatography and spectrometric measurement were used for the quantitative analysis.

#### 2.5.1 Gas chromatography-mass spectrometry (GC-MS) analysis

The MECP was analyzed in a mass spectrometer (TQ 8040, Shimadzu Corporation, Kyoto, Japan) using the electron impact ionization (EI) method and a gas chromatograph (GC-17A, Shimadzu Corporation) with a fused silica capillary column (Rxi-5 ms; 0.25 m film, 30 m long and internal diameter 0.32 mm) coated with DB-1 (J&W). The oven temperature was set at 70 °C (0 min); 10 °C, 150 °C (5 min); 12 °C, 200 °C (15 min); 12 °C, 220 °C (5 min), with a hold time of 10 min. The inlet temperature was 260 °C. The flow rate of the column was 0.6 mL/min helium gas at constant pressure (90 kPa). The GC to MS interface temperature was 280 °C. The MS was used in scanning mode, with a scanning range of 40–350 amu. The ionization mode was electron ionization (EI), and the mass range was 50–550 m/z. One microliter of the sample was injected in the split less injection mode. The total GC-MS run time was 50 min. The compounds in the peak areas were identified by comparison with the national institute of standards and technology (NIST) GC-MS library version 08-S.

#### 2.5.2 Total phenol content (TPC)

The TPC of MECP was evaluated using Folin–Ciocalteu reagent (FCR), according to Reza et al. (2018) (Reza et al., 2018a). One milliliter of FCR was diluted in 9 mL of distilled water; 2.5 mL of diluted FCR and 2.5 mL of Na_2_CO_3_ (20%) was mixed with 500 μg/mL extract. The solution was then filled to 10 mL using distilled water. The solution was incubated for 20 min (25 °C), and the absorbance at 765 nm was observed in triplicate. Gallic acid was utilized as the standard in the TPC determination of milligrams of gallic acid equivalents (GAE) per gram.

#### 2.5.3 Total flavonoid content (TFC)

The TFC of MECP was carried out according to Reza et al. (2018) (Reza et al., 2018a). To measure the TFC, 0.5 mL of extract was mixed with 1.5 mL of methanol, followed by the addition of 0.1 mL of AlCl_3_ (10%), 0.1 mL of CH_3_CO_2_K (1 M), and 2.8 mL of distilled water. The mixture was incubated for 30 min (25 °C), and the absorbance at 415 nm was observed in triplicate. The blank solution contained all the reagents except for the extract. By using quercetin as standard, the TFC estimation was evaluated in terms of milligrams of quercetin equivalents (QE) per gram.

### *2.6* *In vitro* antioxidant activity

#### 2.6.1 DPPH (1, 1-diphenyl-2-picryl-hydrazyl) radical scavenging activity

The scavenging activity of MECP was evaluated using the standard protocol of Braca, (2001). Serial dilutions of the plant extract and ascorbic acid were made at concentrations of 500, 250, 125, and 62.5 μg/mL. Of each dilution, 0.1 mL was added to 3 mL of 0.004% DPPH solution (4 mg DPPH in 100 mL of 95% methanol), followed by incubation for 30 min (25 °C). The absorbance was detected at 517 nm by a ultraviolet (UV) spectrophotometer. The percent radical scavenging activity (%) was calculated as follows (Equation 1):

(1) (%) Radical scavenging = [(A_0_ - A_1_) / A_0_] ×100

where, A_0_ = absorbance of the control and A_1_ = absorbance of the extract.

### *2.7* *In vitro* anti-inflammatory activity

#### 2.7.1 Inhibition of protein denaturation

The anti-inflammatory activity of MECP was evaluated according to a previously reported method with minor modifications (Juvekar et al., 2009; Uddin et al., 2016). Serially diluted concentrations (62.5, 125, 250, and 500 μg/mL) of MECP and diclofenac sodium were obtained. From the dilutions, 0.5 mL was mixed with 0.45 mL of aqueous albumin (5% w/v), and the pH was adjusted to 6.3 using 1 N HCl. The samples were first incubated for 20 min at 37 °C and again for 30 min at 57 °C. After the incubations, the solutions were cooled and then 2.5 mL of phosphate buffer was added. The absorbance was measured by a UV-spectrophotometer (416 nm). The blank solution contained all the reagents except the extract. The percent inhibition protein denaturation was calculated as follows (Equation 2):

(2) (%) Inhibition of protein denaturation = [(A_c_ - A_s_) / A_c_] × 100

where, A_c_ = absorbance of control and A_s_ = absorbance of sample.

### 2.8 Thrombolytic activity

#### 2.8.1 Statement on informed consent of the donors

The volunteer donors were supplied a consent form that informed them of the title of the research project, the names and contact details of the investigators, and the purpose of the research. Additionally included were a brief step-by-step description of the proposed research, the donor inclusion and exclusion criteria, whether donors would receive any therapy or not, the volume of blood to be taken and a statement regarding possible discomfort at the puncture sites, and the time required for the blood sampling. An explanation was given as to whether the research data would be used beyond the current study. There was also a statement discussing the nature of this study group and a caution regarding the limits of confidentiality. The consent form stated that the sample was restricted to this individual study and was not to be used for future research projects. Treatment alternatives and the aims of the research were described. A confidentiality statement was included in the consent form and stated that “confidentiality will be respected and no information that discloses the identity of the participant will be released or published without consent unless required by the law of states”. Finally, detailed contact information (name, area code and phone number) of the investigators was provided in case the donors had any questions about the study. The consent form concluded with questions on the above disclosures in a Yes/No format, followed by a space for the donor's signature (with date) (Emran et al., 2015; Rahman et al., 2013).

#### 2.8.2 Clot lysis

The *in vitro* thrombolytic activity was evaluated according to the method presented by Dewan and Das (2013). The health status of seven male volunteers aged 22–25 years was screened by assessing the body temperature (°C), heartbeat (bpm), blood pressure (mmHg), and BMI immediately before the drawing a sample of venous blood. The health status results are shown in Figure 3(B). The selection criteria also stipulated donors who were non-smokers who did not drink alcohol and who were healthy with no history of cardiovascular or anticoagulant medication use. In total, 3 mL of venous blood was drawn and transferred to a 0.5 mL pre-weighed Eppendorf tube. To form a clot, the tube was incubated for 45 min (37 °C). After clot formation, the released serum was carefully removed without disturbing the clot, and each tube was reweighed to determine the clot weight. One hundred microliters each of standard streptokinase, normal saline, and the methanol extract of *C. pectinata* leaves (MECP) at various concentrations (10, 50, 100, 300 and 500 mg/mL) were added to the tube. The sample was incubated again for 90 min (37 °C). Following the incubation, the acquired fluid was carefully separated and weighed, and the difference between the clot weights was obtained. The percent clot lysis activity was calculated as follows (Equation 3):

(3) (%) Clot lysis = (weight of released clot / clot weight) × 100

### 2.9 Acute toxicity study

An acute oral toxicity study was performed according to the OECD guidelines for the testing of chemicals, Test No. 423 (OECD, 2001). Five animals were used in the toxicity study, and they received a single oral dose of MECP (1000, 2000 or 4000 mg per kg body weight) by oral gavage. After the dose administration, food was withdrawn for 3–4 h. The individual animals were closely observed during the first 30 min after dosing, and periodically for the first 24 h. They were observed for a total of 3 days to record any delayed toxicity. Changes in the skin, fur, eyes, mucous membranes, respiratory and circulatory rates, and autonomic and central nervous system (CNS) function were observed. The median lethal dose was used (LD_50_ > 2.0 g/kg) to calculate the effective therapeutic dose (Rakib et al., 2020).

### 2.10 Experimental design

Male and female Swiss albino mice were separated into control, standard, and test groups of MECP (200 and 400 mg/kg, b.w) included five mice per group. The control group received 1% Tween 80 in water (10 mL/kg, b.w), whereas test groups were administrated MECP at doses of 200 and 400 mg/kg, b.w, respectively by oral gavage. The standard drug diazepam (1 mg/kg, b.w) intraperitoneally (i.p.) was used in open field test, hole-cross test, elevated plus maze test, hole-board test, and light–dark box test, while diclofenac sodium (10 mg/kg b.w, i.p.) was used for the acetic acid-induced writhing inhibition test. The loperamide (5 mg/kg b.w) was administrated for the castor oil-induced diarrhea and gastrointestinal motility test by charcoal marker.

### 2.11 Locomotor activity

#### 2.11.1 Open field test

The sedative-anxiolytic effect of MECP evaluated by using behavioral parameters such as the number of square movements according to a previously described method (Saleem et al., 2011). The open field device is a square box (60 × 60 × 60 cm) with 25 squares of equal dimensions (5 × 5 cm) marked in black and white. Mice were administered as mentioned in section 2.10. Following administration, the mice were individually placed in the apparatus at 0, 30, 60, 90, and 120 min and observed for 3 min.

#### 2.11.2 Hole-cross test

The hole-cross test was performed by a previously described method with modifications (Subhan et al., 2008) using a hole-cross device (30 × 20 × 14 cm) with a partition in the middle and a hole (3 cm in diameter) made at a height of 7 cm. Mice were administered as mentioned in section 2.10. After treatment, the mice were placed individually in the apparatus. The total number of crosses by the hole was recorded for the mice at 0, 30, 60, 90, and 120 min for three minutes.

### 2.12 Anxiolytic activity (*in vivo*)

#### 2.12.1 Elevated plus maze (EPM)

The EPM test was used to evaluate anti-anxiety behavior. The test apparatus was made of two open arms (35 × 5 cm^2^), two closed arms (35 × 20 cm^2^), and a central square (5 × 5 cm^2^). The EPM was elevated 25 cm off the floor. The randomly separated mice were administered as mentioned in section 2.10. At 60 min after treatment, the mice were placed individually in the central square section of the EPM. The observation was recorded for 5 min. The percentage of time spent and entry into open arm was calculated as follows (Equations. (4) and (5)): (Thippeswamy et al., 2011).

(4) $$\left(\%\right)\;Time\;spent\;in\;open\;arm=\frac{time\;spent\;in\;open\;arm}{time\;spent\;in\;open\;arm+time\;spent\;in\;closed\;arm}\;\times 100$$

(5) $$\left(\%\right)\;Entry\;\mathrm{int}o\;open\;arm=\frac{Entry\;\mathrm{int}o\;open\;arm}{Entry\;\mathrm{int}o\;open\;arm\;+entry\;\mathrm{int}o\;closed\;arm}\times 100$$

#### 2.12.2 Hole-board test (HBT)

A hole-board apparatus was used to evaluate the anxiolytic activity. The apparatus consisted of a wooden board (20 cm × 40 cm) with 16 equally spaced holes. The HBT is based on head dipping and measures anxiety behavior with exploratory activity (Sonavane et al., 2002). The randomly separated mice were administered as mentioned in section 2.10. The mice were placed in the center of the apparatus 30 min after the administration. The number of head dipping events and the latency of the first head dipping were recorded for five minutes.

#### 2.12.3 Light–dark box test (LDT)

The anxiolytic activity of MECP was assessed by the light–dark box test according to a previous method with modifications (Moreira et al., 2000). The LDT apparatus comprised a rectangular box (46 × 27 × 30 cm^3^) that was divided into a small area (18 × 27 cm^2^) and a large area (27 × 27 cm^2^) with an opening door (7.5 × 7.5 cm^2^) located in the center of the partition between them. The apparatus also included a light source (400 lx). The randomly separated mice were administered as mentioned in section 2.10. At 30 min after administration, the mice were placed in the dark compartment. The number of transitions and the time spent in the light compartment were measured for 5 min.

### 2.13 Analgesic activity

#### 2.13.1 Acetic acid-induced writhing inhibition test

To investigate the analgesic activity, the acetic acid-induced writhing test was applied (Taur et al., 2011). The dosing of the randomly separated mice was followed as mentioned in section 2.10. Thirty minute after administration, 1% acetic acid (v/v) (10 mL/kg, b.w) was administered intraperitoneally and the writhing time was recorded for 15 min. The percent writhing inhibition (%) was calculated as follows (Equation 6):

(6) (%) Inhibition = [(A - B) / A] × 100

where, A = mean number of writhing in the control group and B = mean number of writhing in the test group.

### 2.14 Anti-diarrheal activities

#### 2.14.1 Castor oil-induced diarrhea

Male and female mice were fasted for 18 h before the experiment. The dosing of the randomly separated mice was followed as mentioned in section 2.10. After 1 h, castor oil (0.5 mL) was administered by oral gavage, and the mice were kept in separate cages that had blotting paper on the floor. The amount of wet and dry feces was noted every hour for four hours. At the start of every hour, the blotting paper was replaced. The percent inhibition (%) of antidiarrheal activity was calculated as follows (Equation 7): (Franca et al., 2008).

(7) (%) Inhibition = [(A - B) / A] × 100

where, A = mean number of diarrheal feces of the control group and B = mean number of diarrheal feces of the treated group.

#### 2.14.2 Gastrointestinal motility test by charcoal marker

Mice were treated as described in the section 2.10. One hour after oral administration, 1 mL of charcoal solution (10% charcoal, 5% gum acacia) was given orally by gavage. The mice were sacrificed 1 h later by chloroform anesthesia, and the total length of the small intestine and the distance traveled by the charcoal marker were assessed and the percent inhibition (%) or antidiarrheal activity was calculated as follows (Equations (8) and (9)): (Adnan et al., 2019).

(8) (%) Inhibition = [(A - B) / A] × 100

where, A = Distance traveled by the charcoal in the control group (cm) and B = Distance traveled by the charcoal in the test group (cm).

(9) Peristalsis index = (Distance traveled by the charcoal / Total length of the small intestine) × 100

### 2.15 *In silico* study

#### 2.15.1 Molecular docking

The major bioactive compounds from the GC-MS analysis were selected for molecular docking based on their biological activity (Table 5). The molecular docking study was conducted according to the previously reported procedures of Sastry et al., (2013) and Tareq (2019) (Sastry et al., 2013; Tareq, 2019). The 3D structures of the following proteins were derived from the Protein Data Bank: COX1 (PDB ID: 2OYE) (Harman et al., 2007), COX2 (PDB ID: 6COX) (Kurumbail et al., 1996), serotonin transporter (PDB ID: 5I6X) (Coleman et al., 2016), potassium channel (PDB ID: 4UUJ) (Lenaeus et al., 2014), tissue plasminogen activator (PDB ID: 1A5H) (Renatus et al., 1997), muscarinic acetylcholine receptor (PDB ID: 4U14) (Thorsen et al., 2014), and PDE4 (PDB ID:4WCU) (Felding et al., 2014). Molecular docking was carried out using Schrödinger Maestro (v11.1).

#### 2.15.2 ADME (absorption, distribution, metabolism, and excretion) analysis

The drug-like properties of the major bioactive compounds of the methanol extract of *C. pectinata* were assessed based on the rule of five, which was described by Lipinski et al., (1997). The rules were: molecular weight, hydrogen bond donor, hydrogen bond acceptor, high lipophilicity (Log p), rotatable bonds, and percentage of human oral absorption. The ADME attributes of compounds were evaluated using the QikProp module (Schrödinger v11.1) (Natarajan et al., 2015).

### 2.16 Statistical analysis

Values were characterized as the mean ± SEM (standard error of the mean). ^∗^*P* < 0.05, ^#^*P* < 0.01 and ^$^ *P* < 0.001 indicated statistical significance compared to the control group according to unpaired t-test (GraphPad Prism version 7.0). The *in vitro* study utilized triplicate measurements, whereas the *in vivo* study included five mice per group.

## 3 Results

### 3.1 Qualitative phytochemical analysis

The phytochemical characteristics of *C. pectinata* are listed in Table 1S. The results showed the presence of active compounds. Alkaloids, carbohydrates, flavonoids, saponins, steroids, cardiac glycosides, glycosides, coumarin, amino acids, resins, gums and mucilage, phytosterols, and sterols were found.

### 3.2 Quantitative phytochemical constituents

#### 3.2.1 GC-MS analysis of *C. pectinata*

**Figure 1:**
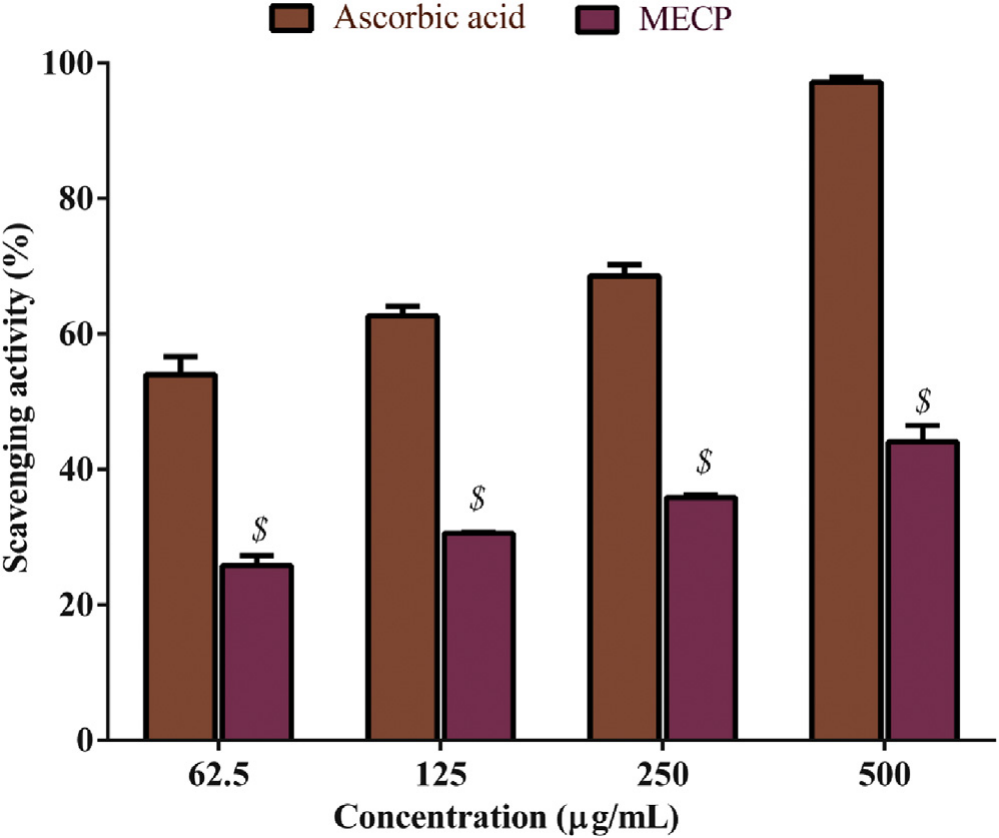
DPPH scavenging activity of methanol extract of *C. pectinata* leaves (MECP) extract compared to the standard ascorbic acid. Values are represented in Mean ± SEM (n = 3). ^$^*P* < 0.001 statistically significant in comparison to Ascorbic acid followed by unpaired t-test (GraphPad Prism 7). MECP: Methanol extract of *Cycas pectinata* leaves.

A total of 25 compounds were eluted between 5.0 and 32 min retention time from the MECP sample (Figure 1S). The compound identification was based on comparisons with the NIST GC-MS library version 08-S (Table 1). The secondary metabolites in the methanol extract with good retention times were identified as androsta-3,5-dien-3-ol, 17-acetyl-3-O-(t-butyldimethylsilyl)-; 2,4,6-cycloheptatrien-1-one, 3,5-bis-trimethylsilyl-; and 2,6-lutidine 3,5-dichloro-4-dodecylthio-, and their retention times were 30.891, 30.230, and 27.856, respectively. Other compounds were phosphonoacetic acid; 3-methylsalicylic acid; sorbitol; phytol; hexadecanoic acid; 15-methyl-, methyl ester; 2,5-dihydroxybenzoic acid; 11,14-eicosadienoic acid, methyl ester; linoleic acid ethyl ester; epinephrine, (beta)-; 1-hexadecane sulfonamide, N-(3-aminopropyl)-; 2- bromopropionic acid, pentadecyl ester; heptadecanoic acid, heptadecyl ester; decanal; quinoline-5,8-dione-6-ol, 7-[[(4-cyclohexylbutyl)amino]methyl]-; 3-chloropropionic acid, 3-tetradecyl ester; hentriacontane-10,14,16-trione; eicosyl isopropyl ether; 13 docosenamide, (Z)-; 9-octadecenamide, (Z)-; cis-11-eicosenamide; 3,4-dihydroxymandelic acid; and tricyclo[4.2.1.0(2,5)]non-7-ene, 3,4-di(tris(trimethylsilyloxy)silyl)-.

**Table 1:** Quantitative compounds identified from methanol extract of *C. pectinata* by GC-MS analysis.

SL No.	Retention time	Molecular formula	Compound name	Nature
1.	5.881	C_2_H_5_O_5_P	Phosphonoacetic Acid	Organophosphorus
2.	8.056	C_8_H_8_O	3-Methylsalicylic acid	Phenol
3.	10.351	C_6_H_14_O	Sorbitol	Sugar alcohol
4.	12.516	C_20_H_40_O	Phytol	Diterpene
5.	13.450	C_18_H_36_O_2_	Hexadecanoic acid, 15-methyl-,methyl ester	Terpenoid
6.	14.889 and 10.087	C_7_H_6_O_4_	2,5-Dihydroxybenzoic acid	Benzoic acid
7.	15.170	C_21_H_38_O_2_	11,14-Eicosadienoic acid, methyl ester	Terpenoid
8.	15.170	C_20_H_36_O_2_	Linoleic acid ethyl ester	Fatty acid
9.	16.199	C_9_H_13_NO_3_	Epinephrine, (.beta.)-	Alkaloid
10.	17.623	C_7_H_18_N_4_O_2_S	1-hexadecane sulfonamide, N-(3-aminopropyl)-	Alkaloid
11.	20.009	C_18_H_35_BrO_2_	2- Bromopropionic acid, pentadecyl ester	Ester
12.	20.009	C_34_H_68_O_2_	Heptadecanoic acid, heptadecyl ester	Saturated fatty acid
13.	20.360	C_10_H_20_O	Decanal	Aldehyde
14.	20.360	C_20_H_26_N_2_O_3_	Quinoline-5,8-dione-6-ol, 7-[[(4-cyclohexylbutyl)amino]methyl]-	Amines/aniline
15.	20.360	C_17_H_33_ClO_2_	3-Chloropropionic acid, 3-tetradecyl ester	Ester
16.	24.033	C_31_H_58_O_3_	Hentriacontane-10,14,16-trione	Fatty acid
17.	24.033	C_23_H_48_O	Eicosyl isopropyl ether	Ether
18.	24.525	C_22_H_43_NO	13-Docosenamide, (Z)-	Amide
19.	24.525	C_18_H_35_NO	9-Octadecenamide, (Z)-	Oleic acid
20.	24.525	C_20_H_39_NO	cis-11-Eicosenamide	Amide
21.	26.385, 14.889 and 11.861	C_8_H_8_O_5_	3,4-Dihydroxymandelic acid	Mandelic acid
22.	27.856	C_27_H_64_O_6_Si_8_	Tricyclo[4.2.1.0(2,5)]non-7-ene, 3,4-di(tris(trimethylsilyloxy)silyl)-	Organic compound
23.	27.856	C_19_H_31_Cl_2_NS	2,6-Lutidine 3,5-dichloro-4-dodecylthio-	Heterocyclic
24.	30.230, 26.385 and 25.827	C_13_H_22_OSi_2_	2,4,6-Cycloheptatrien-1-one, 3,5-bis-trimethylsilyl-	Ketone
25.	30.891 and 17.623	C₂₇H₄₄O_2_Si	Androsta-3,5-dien-3-ol, 17-acetyl-3-O-(t-butyldimethylsilyl)-	Steroids

#### 3.2.2 Total phenol and flavonoid content

The total phenol and total flavonoid contents were estimated using the regression equations for gallic acid (y = 0.0039x + 0.0406; R^2^ = 0.9981) and quercetin (y = 0.0102x - 0.0637; R^2^ = 0.9693), respectively (Table 2). The total phenol content in MECP was higher (209.85 ± 3.40 mg GAE/g dry extract) than the total flavonoid content (105.17 ± 3.45 mg QE/g dry extract).

**Table 2:** Quantitative analysis of antioxidant relevant phytochemicals total phenol content, total flavonoid content of methanol extract of *C. pectinata* leaves with IC_50_ value.

Treatment	Total phenol content (mg GAE/g extract)	Total flavonoid content (mg QE/g extract)	IC_50_ (μg/mL)
MECP	209.85 ± 3.40	105.17 ± 3.45	631.44
Ascorbic acid	--	--	19.08

MECP: Methanol extract of *Cycas pectinata* leaves*.*

### 3.3 *In vitro* antioxidant activity

#### 3.3.1 DPPH radical scavenging assay

MECP exhibited a moderate radical scavenging activity in the DPPH assay. The minimum inhibitory concentration (IC_50_) value of MECP was 631.44 μg/mL, where the regression equation was y = 0.0401x + 24.679; R^2^ = 0.9764. The standard ascorbic acid IC_50_ value was 19.08 μg/mL, and the regression equation was y = 0.0957x + 48.174; R^2^ = 0.9807 (Table 2). The antioxidant DPPH scavenging activity of MECP was significantly lower than that of ascorbic acid. Among all four concentrations of MECP, 500 μg/mL showed the highest scavenging activity (44.09%, *P* < 0.001), while this concentration of ascorbic acid exhibited 97.17% scavenging activity (Figure 1).

### 3.4 Anti-inflammatory activity (in vitro)

#### 3.4.1 Protein denaturation assay

**Figure 2:**
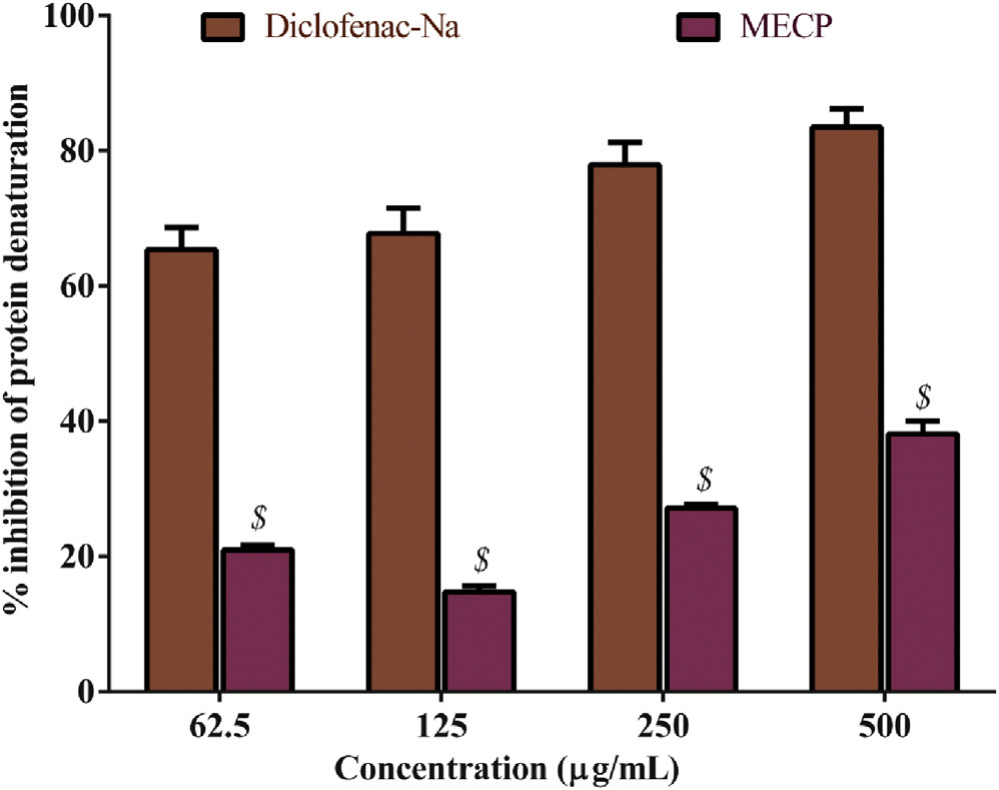
Percentage inhibition of protein denaturation of methanol extract of *C. pectinata* leaves (MECP) extract compared to the standard diclofenac sodium. Values are represented in Mean ± SEM (n = 3). ^$^*P* < 0.001 statistically significant in comparison to diclofenac sodium followed by unpaired t-test (GraphPad Prism 7). MECP: Methanol extract of *Cycas pectinata* leaves.

MECP and diclofenac sodium exhibited significant (*P* < 0.001) dose-dependent inhibition in the protein denaturation assay, as shown in Figure 2. The 500 μg/mL MECP showed 38.12% of the maximum protein denaturation inhibition, whereas diclofenac sodium showed 83.50% inhibition.

### 3.5 Thrombolytic activity

**Figure 3:**
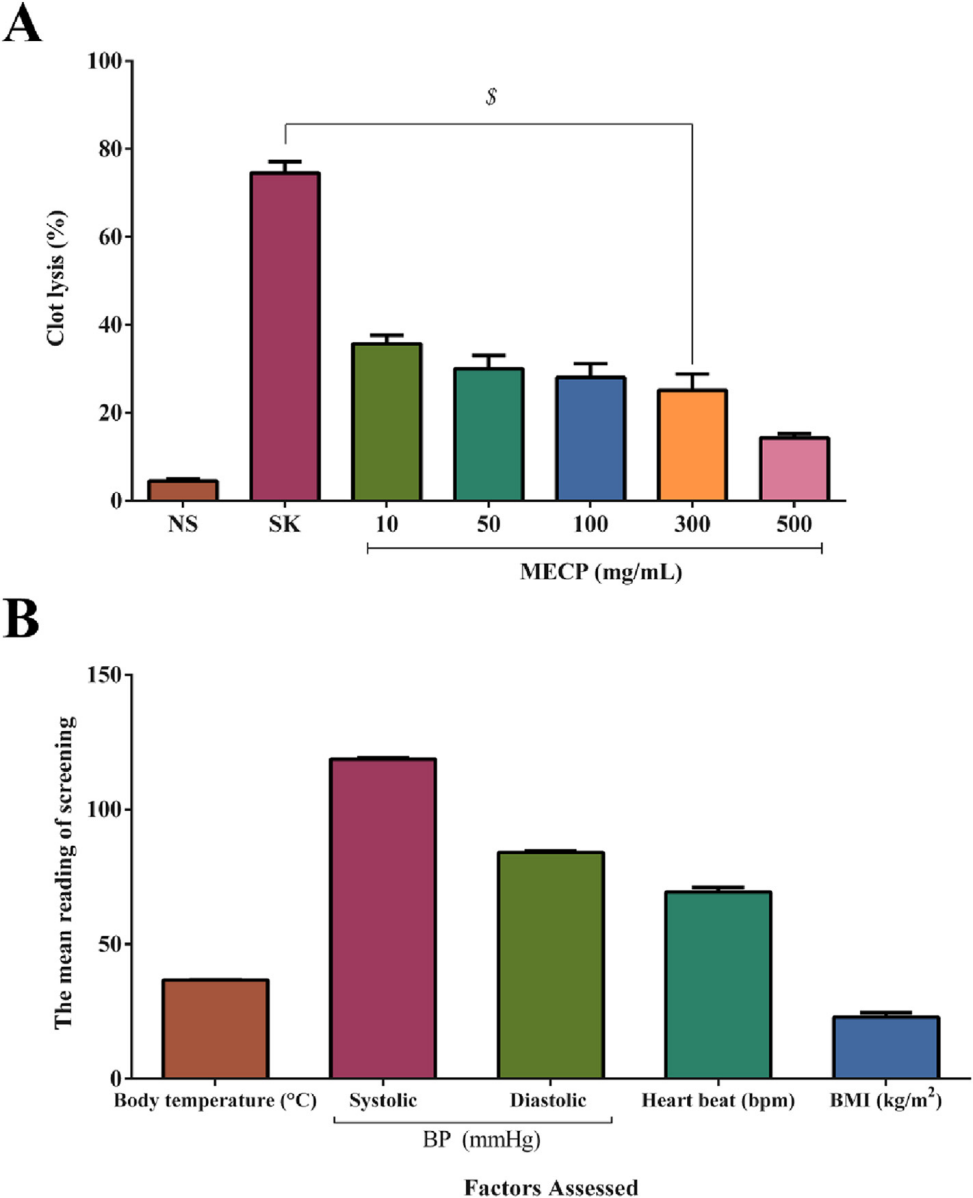
(A) The clot lysis activity of methanol extract of *C. pectinata* leaves (MECP) extract, streptokinase (SK) and normal saline (control). (B) The healthy volunteer status presented. Values are represented in Mean ± SEM. ^$^*P* < 0.001 statistically significant in comparison to normal saline (control) followed by unpaired t-test (GraphPad Prism 7). MECP: Methanol extract of *Cycas pectinata* leaves.

The clot lysis activity and the status of the healthy volunteers are respectively presented in Figure 3 (A, B). The plant extract (10, 50, 100, 300, and 500 mg/mL) showed a moderate clot lysis ability, and the 10 mg/mL concentration showed 35.72% (*P* < 0.001) clot lysis activity. The positive control streptokinase exhibited 74.52% clot lysis activity, while normal saline showed only a minor amount of clot lysis (4.49%).

### 3.6 Acute toxicity study

The MECP extract did not result in morbidity, mortality, or any adverse behavioral changes during the 8 h and 3 day observation periods after the oral administration of 1000, 2000, and 4000 mg/kg.

### 3.7 Locomotor activity

#### 3.7.1 Open field test

**Figure 4:**
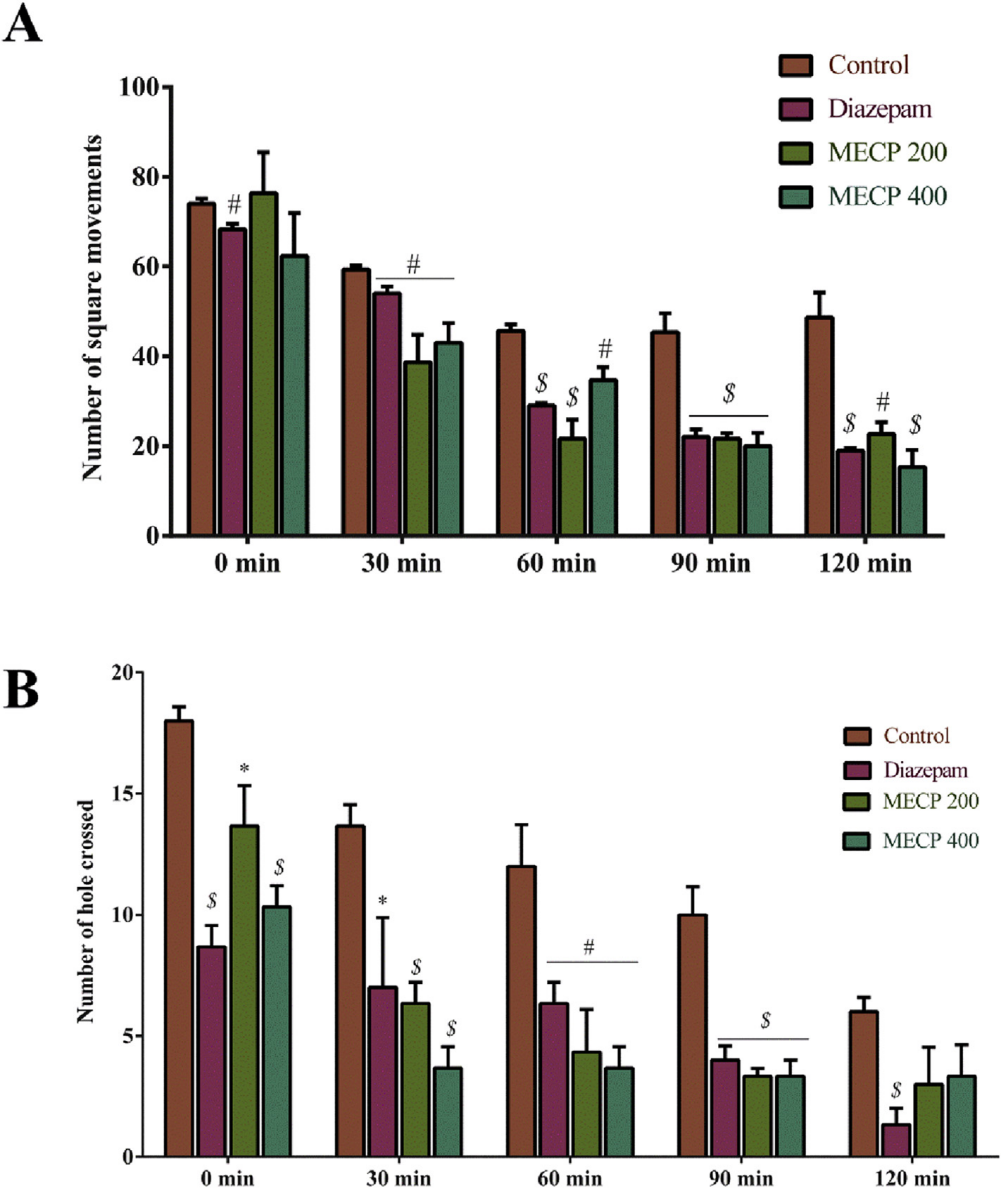
(A) Locomotor activity of methanol extract of *C. pectinata* leaves and diazepam on open field test. **(**B**)** Locomotor activity of methanol extract of *C. pectinata* leaves and diazepam on hole-cross test. Values are represented in Mean ± SEM (n = 5). ^∗^*P* < 0.05, ^#^*P* < 0.01 and ^$^*P* < 0.001 statistically significant in comparison to control followed by unpaired t-test (GraphPad Prism 7). MECP: Methanol extract of *Cycas pectinata* leaves.

MECP (200 and 400 mg/kg) elicited a dose-dependent effect on locomotor activity, and the effects were statistically significant (*P* < 0.05) in comparison to the 1% Tween-80 control group. CNS activity was apparent owing to the decrease in the square movement from the initial (0 min) to the final observation (120 min). The 400 mg/kg dose revealed a significant reduction in number of square movements over the 0–120 min observation period as follows: 62.33 ± 9.53, 43.0 ± 4.36, 34.67 ± 2.91, 20.0 ± 3.0, and 15.33 ± 3.84, whereas the standard drug diazepam showed values of 68.33 ± 1.20, 54.0 ± 1.53, 29.0 ± 0.58, 22.0 ± 1.70, and 19.0 ± 0.58 (Figure 4A).

#### 3.7.2 Hole-cross test

The CNS effect was evaluated by assessing locomotion in mice. The holes crossed by the mice decreased from the first observation (0 min), as confirmed by the reduction of hole crossings compared to the 1% Tween-80 control group (Figure 4B). The treatment doses (200 and 400 mg/kg) showed a significant dose-dependent (*P* < 0.05) locomotor effect. The 400 mg/kg dose demonstrated a significant reduction over the 0–120 min observation period (10.33 ± 0.88, 3.67 ± 0.88, 3.67 ± 0.88, 3.33 ± 0.67, and 3.33 ± 1.30), whereas the standard drug diazepam showed values of 8.67 ± 0.88, 7.0 ± 2.89, 6.33 ± 0.88, 4.0 ± 0.58, and 1.33 ± 0.67.

### 3.8 Anxiolytic activity

#### 3.8.1 Elevated plus maze test

**Figure 5:**
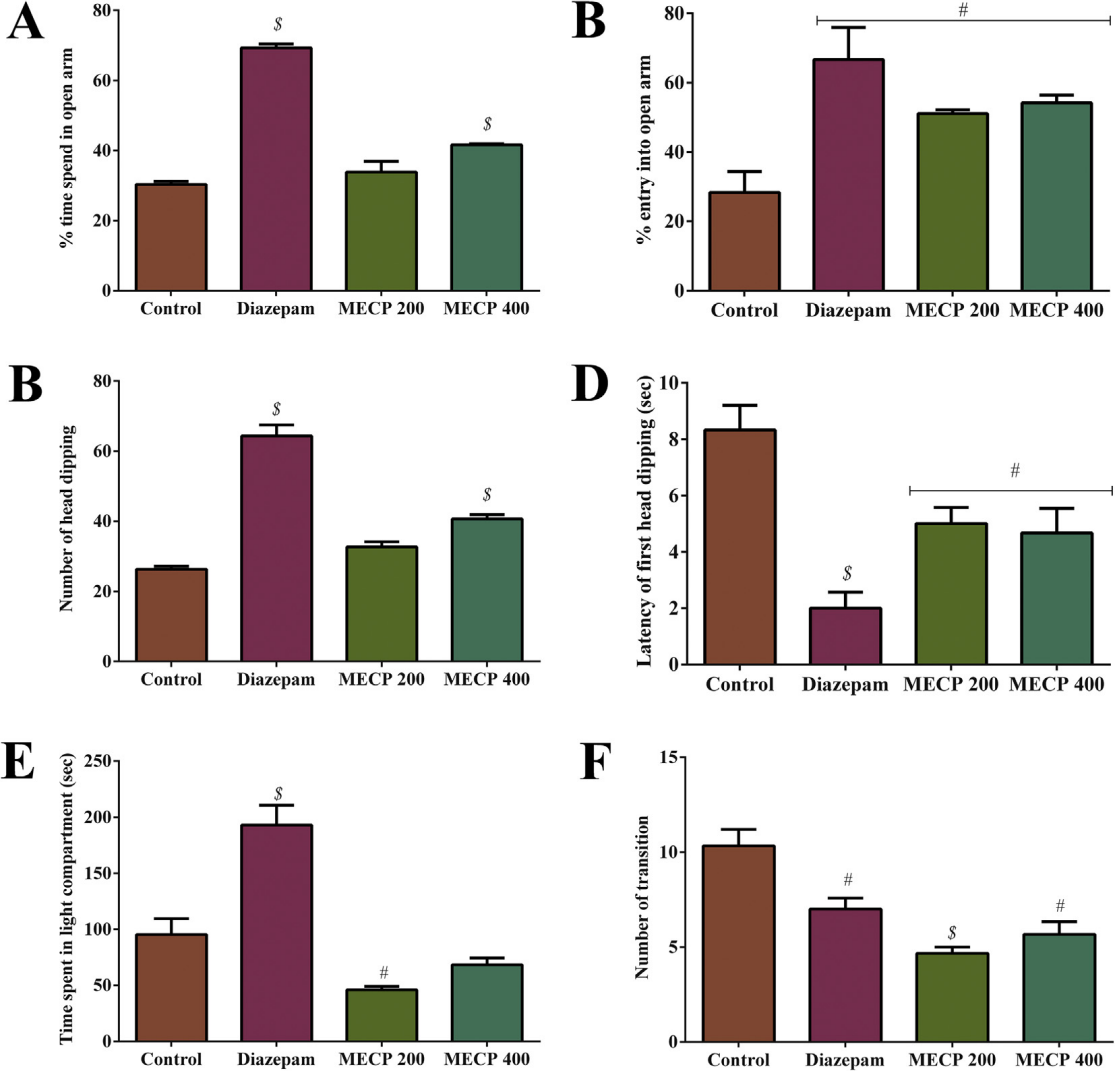
(A) Anxiolytic activity of methanol extract of *C. pectinata* leaves and diazepam on % of time spend in open arm in elevated plus maze test. (B) Effect of methanol extract of *C. pectinata* leaves and diazepam on % of entry in open arm in elevated plus maze test. (C) Anxiolytic activity of methanol extract of *C. pectinata* leaves and diazepam on number of head-dipping in hole-board test. (D) Effect of methanol extract of *C. pectinata* leaves and diazepam on latency of first head-dipping in hole-board test. (E) Anxiolytic activity of methanol extract of *C. pectinata* leaves and diazepam on time spent in light compartment in light-dark test. (F) Effect of methanol extract of *C. pectinata* leaves and diazepam on number of transition in light-dark test. Values are represented in Mean ± SEM (n = 5). ^#^*P* < 0.01 and ^$^*P* < 0.001 statistically significant in comparison to control followed by unpaired t-test (GraphPad Prism 7). MECP: Methanol extract of *Cycas pectinata* leaves.

The MECP treatment increased the percentage of time spent and entry into the open arms, as shown in Figure 5 (A, B). MECP led to a significant percentage of time spent in the open arms, which increased to 33.87 ± 3.06 and 41.65 ± 0.31 (*P* < 0.001) for the 200 and 400 mg/kg doses, respectively, while diazepam showed 69.33 ± 1.15 (*P* < 0.001). The 400 mg/kg MECP and 1 mg/kg diazepam treatments were statistically significant compared to the control (30.33 ± 0.88). Additionally, the 200 and 400 mg/kg MECP treatments led to a significant percentage of entry into open arms (51.11 ± 1.11 and 54.23 ± 2.17, respectively (*P* < 0.01)), whereas diazepam showed 66.67 ± 9.27.

#### 3.8.2 Hole-board test

In the hole-board test, the amount of head dipping increased with increasing dose and is summarized in Figure 5 (C, D). The 400 mg/kg MECP treatment significantly (*P <* 0.001) increased the amount of head dipping (40.67 ± 1.20) compared to the control (26.33 ± 0.88), whereas the diazepam treatment showed 64.33 ± 3.16 (*P <* 0.001). Furthermore, the latencies of head dipping observed for the MECP treatments (200 and 400 mg/kg) were 5.0 ± 0.58 and 4.67 ± 0.88 (*P* < 0.01), whereas this was 2.0 ± 0.57 (*P* < 0.001) for diazepam.

#### 3.8.3 Light–dark box test

In the light–dark box experiment, the mice exhibited a significant amount of transitions to the light compartment. The 200 and 400 mg/kg MECP treatments led to 4.67 ± 0.33 and 5.67 ± 0.67 times the number of transitions to the light compartment (diazepam; 7.0 ± 0.58). The results are presented in Figure 5 (E, F) Additionally, the 400 mg/kg MECP led to the highest time spent in the light compartment, while the 200 mg/kg MECP treatment led to significant (*P* < 0.01) activity in the light compartment.

### 3.9 Analgesic activity

#### 3.9.1 Acetic acid-induced writhing test

**Figure 6:**
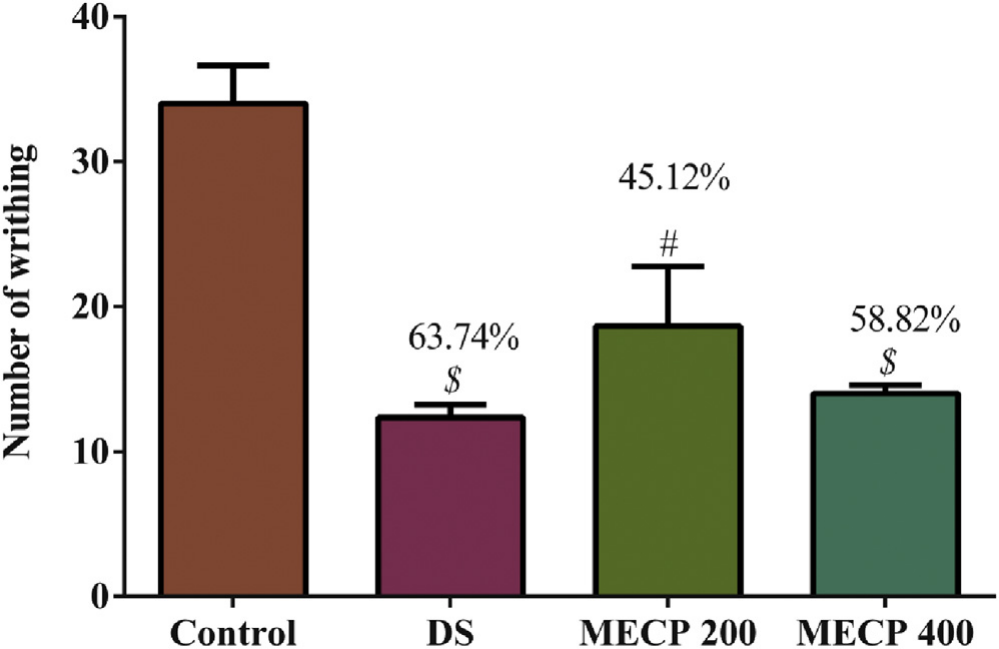
Analgesic activity of methanol extract of *C. pectinata* leaves and diclofenac sodium (DS) on acetic acid-induced writhing test with percentage of inhibition of analgesia. Values are represented in Mean ± SEM (n = 5). ^#^*P* < 0.01 and ^$^*P* < 0.001 statistically significant in comparison to control followed by unpaired t-test (GraphPad Prism 7). MECP: Methanol extract of *Cycas pectinata* leaves; DS: Diclofenac sodium.

MECP treatment led to significant antinociceptive activity, as shown in Figure 6. Dose-dependent analgesia was observed for the tested doses. The 400 mg/kg MECP dose led to a 58.82% (*P* < 0.001) reduction in writhing, whereas the positive control diclofenac sodium showed a 63.74% reduction. The 200 mg/kg MECP dose also significantly (*P* < 0.01) inhibited writhing (45.12%).

### 3.10 Antidiarrheal activity

#### 3.10.1 Castor oil-induced diarrhea

MECP treatment led to a significant dose-dependent inhibition of diarrhea and defecation, as summarized in Table 3. The tested dose of 400 mg/kg elicited the highest percentage of defecation (45.21%) and diarrhea (42.71%) inhibition, whereas the standard drug loperamide led to a 63.01% and 65.63% inhibition in defecation and diarrhea, respectively.

**Table 3:** The effect of *C. pectinata* on castor oil induced diarrhea in mice (feces count).

Treatment (mg/kg)	Total number of feces	% of Inhibition of defecation	Total number of diarrheal feces	% Inhibition of diarrhea
Control	14.60 ± 0.87	-	6.40 ± 0.81	-
Loperamide (5)	5.40 ± 0.24 ^*$*^	63.01	2.20 ± 0.20 ^*$*^	65.63
MECP 200	11 ± 0.58 ^*#*^	24.66	5.33 ± 0.33	16.67
MECP 400	8 ± 1.53 ^*#*^	45.21	3.67 ± 0.33 ^*#*^	42.71

Values are represented in Mean ± SEM (n = 5). ^#^*P* < 0.01, ^$^*P* < 0.001 are statistically significant in comparison to Tween-80 (Control) followed by unpaired t-test (GraphPad Prism 7). MECP: Methanol extract of *Cycas pectinata* leaves*.*

#### 3.10.2 Gastrointestinal motility (charcoal marker)

MECP led to a significant decrease in the peristalsis index; 400 mg/kg MECP led to a significant reduction (53.16 ± 1.50; *P < 0.01*), whereas the standard drug loperamide showed a 44.20 ± 1.96% (*P* < 0.001) decrease. The 200 mg/kg MECP treatment dose also significantly decreased the peristalsis index (59.16 ± 2.31, *P < 0.01*). The 400 mg/kg dose of MECP elicited the highest percentage (27.5%) of intestinal motility inhibition, while the standard drug loperamide showed 45% inhibition (Table 4).

**Table 4:** The effect of *C. pectinata* on gastrointestinal motility by charcoal marker.

Treatment (mg/kg)	Total Length of Intestine (cm)	Distance Travel by Charcoal (cm)	Peristalsis Index (%)	% Inhibition
Control (0.1ml/mouse)	48 ± 0.57	40 ± 2.88	83.42 ± 6.49	--
Loperamide (5)	49.66 ± 1.20	22 ± 1.52 ^*$*^	44.20 ± 1.96 ^*$*^	45
MECP 200	59 ± 2.08 ^*$*^	35 ± 2.51	59.16 ± 2.31^*#*^	12.5
MECP 400	54.66 ± 2.33 *^∗^*	29 ± 0.57 ^*#*^	53.16 ± 1.50 ^*#*^	27.5

Values are represented in Mean ± SEM (n = 5). ^∗^*P* < 0.05, ^#^*P* < 0.01, ^$^*P* < 0.001 statistically significant in comparison to Tween-80 (Control) followed by unpaired t-test (GraphPad Prism 7). MECP: Methanol extract of *Cycas pectinata* leaves*.*

### 3.11 *In silico* molecular docking study

In the molecular docking evaluation, 10 major bioactive constituents were selected on the basis of their biological activities. These constituents are summarized in Table 5.

**Table 5:** Biological activity of the selected compounds from GC-MS of *C. pectinata.*

Name	Biological activity	References
3-Methylsalicylic acid	Thrombolytic activity	(Chen, 2019)
2,5-Dihydroxybenzoic acid	Antioxidant, anti-inflammatory	(Kalinowska et al., 2018)
Sorbitol	Diarrhea induced or laxative	(Liauw and Saibil, 2019)
Phytol	Antinociceptive, antioxidant, Anti-inflammatory	(Santos et al., 2013)
Hexadecanoic acid, 15-methyl-, methyl ester	Antioxidant	(Zayed and Samling, 2016)
11,14-Eicosadienoic acid, methyl ester	Anti-inflammatory, anti-oxidant, anti-coronary.	(Gomathi Rajashyamala and Elango, 2015)
Linoleic acid ethyl ester	Antiarthritic and Anti-coronary	(Sudha et al., 2013)
Decanal	Antioxidant	(Liu et al., 2012)
13-Docosenamide, (Z)-	Antinociceptive and anti-inflammatory	(Shareef et al., 2016)
3,4-Dihydroxymandelic acid	Antioxidant, noradrenaline	(Ley et al., 2002)

#### 3.11.1 Molecular docking related to anti-inflammatory activity

**Figure 7:**
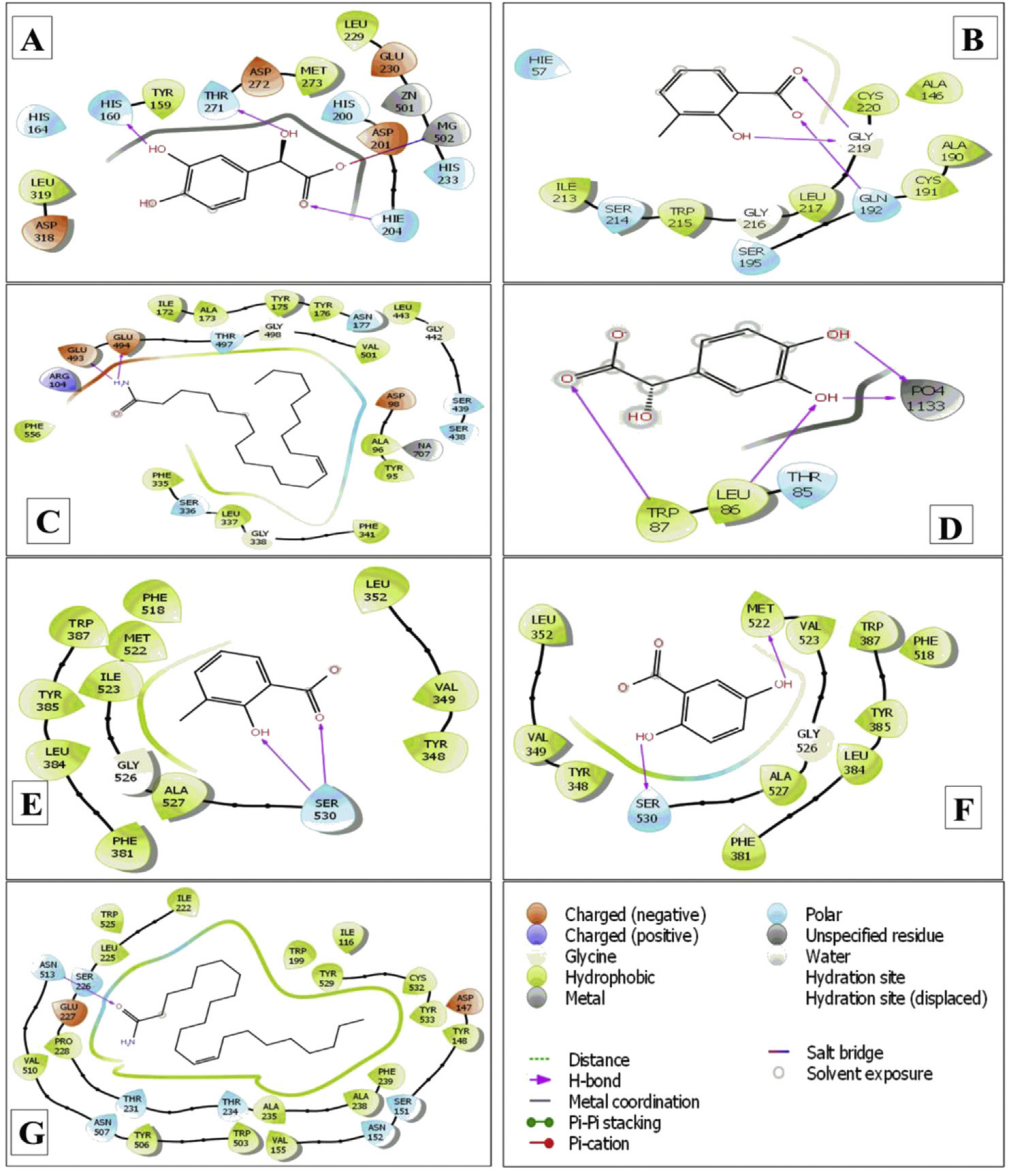
2D representation of best docking score compounds: (A) 3,4-Dihydroxymandelic acid- 4WCU, (B) 3-Methylsalicylic acid-1A5H, (C) 13-Docosenamide, (Z)--5I6X, (D) 3,4-Dihydroxymandelic acid--4UUJ, (E) 3-Methylsalicylic acid--2OYE, (F) 2,5-Dihydroxybenzoic acid--6COX and (G)13-Docosenamide, (Z)--4U14.

The molecular docking results related to anti-inflammatory activity are summarized in Table 6, and the compounds with the best docking scores are shown in Figure 7. Phosphodiesterase-4 inhibitor (PDE4) receptor (PDB ID: 4WCU) was used for this molecular docking simulation, and 3,4-dihydroxymandelic acid showed the strongest docking interaction (-9.079 kcal/mol), followed by 2,5-dihydroxybenzoic acid (-8.996 kcal/mol), 3-methylsalicylic acid (-8.27 kcal/mol), and linoleic acid ethyl ester (-7.014 kcal/mol). The reference drug diclofenac sodium had a docking interaction of -7.836 kcal/mol, which was weaker than that of 3,4-dihydroxymandelic acid. 3,4-Dihydroxymandelic acid interacted with His 160, Thr 271, and Hie 204 via three H-bonds and one salt bridge with MG 502.

**Table 6:** Molecular docking study of major bioactive compounds.

Name	Docking score (kcal/mol)						
	4WCU	1A5H	5I6X	4UUJ	2OYE	6COX	4U14
3-Methylsalicylic acid	-8.27	**-6.292**	-4.752	-3.733	**-6.982**	-6.111	-6.651
2,5-Dihydroxybenzoic acid	-8.996	-6.137	-5.229	-	-6.167	**-6.721**	-6.658
Sorbitol	-4.428	-4.402	-4.382	-4.259	-4.405	-4.306	-3.948
Phytol	-4.177	-1.103	-2.985	-0.902	-4.022	-2.617	-3.77
Hexadecanoic acid, 15-methyl-, methyl ester	-1.725	-0.657	-0.998	1.008	-2.269	-0.465	-2.918
11,14-Eicosadienoic acid, methyl ester	-5.742	-3.29	-5.896	-3.287	-6.243	-5.468	-6.663
Linoleic acid ethyl ester	-7.014	-3.848	-4.945	-2.59	-6.271	-4.794	-6.733
Decanal	0.932	1.867	1.022	2.83	0.439	0.112	0.435
13-Docosenamide, (Z)-	-6.379	-3.519	**-6.4**	-3.349	-5.631	-5.475	**-6.883**
3,4-Dihydroxymandelic acid	**-9.079**	-6.279	-5.804	**-5.104**	-6.29	**-6.413**	-6.386
Reference drugs DS/SK/Diazepam/DS/Loperamide	-7.836	-6.173	-7.956	-3.035	-6.917	-7.545	-8.152

Bold text indicates the best docking score. 4WCU- PDB ID for anti-inflammatory activity, 1A5H- PDB ID for thrombolytic activity, 4UUJ- PDB ID for anxiolytic activity, 5I6X- PDB ID for antidepressant/locomotor activity, 2OYE and 6COX - PDB ID for analgesic activity, 4U14- PDB ID for antidiarrheal activity. DS: Diclofenac sodium, SK: Streptokinase.

#### 3.11.2 Molecular docking related to thrombolytic activity

The molecular docking results related thrombolytic activity was summarized in Table 6, and the compounds with the best docking scores are shown in Figure 7. Tissue plasminogen activator receptor (PDB ID: 1A5H) was used for this molecular docking simulation. 3-Methylsalicylic acid showed the strongest docking interaction (-6.292 kcal/mol), which was stronger than that of the reference drug streptokinase (-6.173 kcal/mol). The other two compounds, 3,4-dihydroxymandelic acid (-6.279 kcal/mol) and 2,5-dihydroxybenzoic acid (-6.137 kcal/mol), showed stronger interactions than streptokinase. 3-Methylsalicylic acid interacted via two H-bonds with Gly 219 and with Gln 192 via one H-bond.

#### 3.11.3 Molecular docking related to locomotor activity

Human serotonin transporter receptor (PDB ID: 5I6X) was used for this molecular docking simulation. The interaction results are summarized in Table 6, and the compounds with the best docking scores are shown in Figure 7. 13-Docosenamide, (Z)- showed the strongest binding affinity, displaying a docking score of -6.4 kcal/mol (reference drug diazepam, -7.956 kcal/mol). 13-Docosenamide, (Z)- interacted with the pocket formed by Glu 493 and Glu 494 via two H-bonds.

#### 3.11.4 Molecular docking related to anxiolytic activity

The molecular docking results related to anxiolytic activity were summarized in Table 6, and the compounds with the best docking scores are shown in Figure 7. The potassium channel receptor (PDB ID: 4UUJ) was used in this molecular docking simulation. 3,4-Dihydroxymandelic acid showed the strongest docking interaction (-5.104 kcal/mol), which was stronger than that of the reference drug diazepam (-3.035 kcal/mol). The compounds sorbitol (-4.259 kcal/mol), 3-methylsalicylic acid (-3.733 kcal/mol), 13-docosenamide, (Z)- (-3.349 kcal/mol), and 11,14-eicosadienoic acid, methyl ester (-3.287 kcal/mol) also showed stronger interactions than diazepam. However, 2,5-dihydroxybenzoic acid did not interact with the pocket of the potassium channel receptor. 3,4-Dihydroxymandelic acid interacted with PO4-1133 via two H-bonds and via two H-bonds with Leu 86 and Trp 87.

#### 3.11.5 Molecular docking related to analgesic activity

The COX1 (PDB ID: 2OYE) and COX2 (PDB ID: 6COX) receptors were utilized for the molecular docking simulations to investigate the analgesic potential. The results are summarized in Table 6, and the best docking scores are shown in Figure 7. For COX1, 3-methylsalicylic acid exhibited the lowest docking score (-6.982 kcal/mol), while diclofenac sodium showed -6.917 kcal/mol. The other compounds with similar docking scores were 3,4-dihydroxymandelic acid (-6.29 kcal/mol), linoleic acid ethyl ester (-6.271 kcal/mol), 11,14-eicosadienoic acid, methyl ester (-6.243 kcal/mol), and 2,5-dihydroxybenzoic acid (-6.167 kcal/mol). For COX2, 2,5-dihydroxybenzoic acid (-6.721 kcal/mol) exhibited the strongest binding affinity, followed by 3,4-dihydroxymandelic acid (-6.413 kcal/mol) and 3-methylsalicylic acid (-6.111 kcal/mol). The reference drug diclofenac sodium exhibited a lower docking score of -7.545 kcal/mol.

#### 3.11.6 Molecular docking related to antidiarrheal activity

The molecular docking results related to the antidiarrheal activity were summarized in Table 6, and the compounds with the best docking scores are displayed in Figure 7. In this molecular docking simulation, the muscarinic acetylcholine receptor (PDB ID: 4U14) was used, and 13-docosenamide, (Z) had the lowest docking score (-6.883 kcal/mol). The reference drug loperamide interacted through one H-bond with Tyr 148 (-8.152 kcal/mol). Linoleic acid ethyl ester also interacted and showed a strong binding affinity (-6.733 kcal/mol). The strong binding affinity of 13-docosenamide, (Z) occurred via one H-bond with Asn 513.

### 3.12 In silico ADME analysis

The drug-like activities were evaluated by ADME analysis using QikProp (Schrödinger v11.1). The ADME properties of the following compounds were assessed: 3-methylsalicylic acid; 2,5-dihydroxybenzoic acid; sorbitol; 3,7,11,15-tetramethyl-2-hexadecen-1-ol; hexadecanoic acid, 15-methyl-, methyl ester; 11,14-eicosadienoic acid, methyl ester; linoleic acid ethyl ester; decanal; 13-docosenamide, (Z)-; and 3,4-dihydroxymandelic acid. 3-Methylsalicylic acid; 2,5-dihydroxybenzoic acid; decanal; and 3,4-dihydroxymandelic acid followed Lipinski's rule of five, while all other compounds violated one rule (Table 7).

**Table 7:** ADME properties of methanol extract of *C. pectinata* by QikProp.

Name	MW^1^	HBD^2^	HBA^3^	Log P^4^	ROTB^5^	PHOA^6^	RO5V^7^
3-Methylsalicylic acid	152.15	2	3	2.9	1	75.399	0
2,5-Dihydroxybenzoic acid	154.12	3	4	1.6	1	58.745	0
Sorbitol	182.17	6	6	-3.1	5	30.771	1
Phytol	296.5	1	1	8.2	13	100	1
Hexadecanoic acid, 15-methyl-, methyl ester	284.5	0	2	8.2	15	100	1
11,14-Eicosadienoic acid, methyl ester	322.5	0	2	8	17	100	1
Linoleic acid ethyl ester	308.506	0	2	7.3	16	100	1
Decanal	156.26	0	1	3.8	8	100	0
13-Docosenamide, (Z)-	337.6	1	1	8.8	19	100	1
3,4-Dihydroxymandelic acid	184.15	4	5	-0.6	2	44.236	0

1 Molecular weight, MW (acceptable range: < 500 g/mol).

2 Hydrogen bond donor, HBD (acceptable range: ≤5).

3 Hydrogen bond acceptor, HBA (acceptable range: ≤10).

4 High lipophilicity (LogP, acceptable range: <5).

5 Rotatable bond, ROTB (≤10).

6 % Human oral absorption.

7 Rule of five violations.

## 4 Discussion

In the field of drug discovery, the role of medicinal plants is continually increasing due to their unique compounds that have various activities to treat different diseases. Globally, 80% of the population directly or indirectly depends on herbal or traditional medicines. The phytochemical analysis of plant extracts has identified compounds that might be responsible for their physiological and therapeutic activities (Majid et al., 2015). The current qualitative study of a plant extract demonstrated the presence of alkaloids, carbohydrates, flavonoids, saponins, steroids, cardiac glycosides, glycosides, coumarin, amino acids, resins, gums and mucilage, phytosterols, and sterols. In the quantitative analysis of the methanol extract of *C. pectinata*, 25 compounds were found by GC-MS between 5.0 and 35.0 min retention time. The majority of the compounds were terpenoids, followed by fatty acids, alkaloids, alcohols, phenols, steroids, and esters.

Antioxidants are very important substances that help to protect the body from free radical damage. ROS including free radicals and RNS are constantly produced during cell metabolism. Free radicals are vital for the functioning of gene expression and receptor activation. However, increased levels of free radicals in the body can become toxic to the biological system and are implicated in several conditions, such as cancer, neurodegenerative disorders, aging, and inflammation (Diaz et al., 2012). The antioxidants contained in plants could help to alleviate free radical generation and other oxidative diseases. Flavonoid and phenolic substances greatly contribute to minimizing oxidation (Reza et al., 2018a). In our study, the total phenol content was present in the highest amount (209.85 ± 3.40 mg GAE/g dry extract), followed by the total flavonoid content (105.17 ± 3.45 mg QE/g dry extract). In the DPPH scavenging assay, the maximum scavenging activity was 44.09% at 500 μg/mL, with a low IC_50_ value. There was a good correlation between the scavenging and the total phenol content due to the presence of phenolic bioactive compounds, such as 3-methylsalicylic acid and 2,5-dihydroxybenzoic acid. phytol; 2,5-dihydroxybenzoic acid; hexadecanoic acid 15-methyl-, methyl ester; 11,14-eicosadienoic acid, methyl ester; decanal; and 3,4-dihydroxymandelic acid are reported to have antioxidant activity, which might be responsible for the significant antioxidant ability of MECP (Gomathi Rajashyamala and Elango, 2015; Kalinowska et al., 2018; Ley et al., 2002; Liu et al., 2012; Santos et al., 2013; Zayed and Samling, 2016).

Physical injuries, heat, microbial diseases, and toxic biochemical irritations can all cause inflammation, which is a reaction process that occurs in living tissues. The reaction of cells to inflammation will prompt a definite pathological condition, categorized by redness, heat, swelling, and soreness, and it can reduce physiological capacities. Inflammation is involved in the pathogenesis of numerous diseases, while the denaturation of intracellular proteins can lead to tissue injury. The ability of a substance to inhibit protein denaturation signifies the potential for anti-inflammatory activity (Osman et al., 2016; Ricciotti and FitzGerald, 2011). The anti-inflammatory effects of MECP (500 μg/mL) exhibited a significant (*P* < 0.001) inhibition of protein denaturation (38.12%), whereas diclofenac sodium exhibited 83.50% inhibition. 2,5-Dihydroxybenzoic acid; phytol; 11,14-eicosadienoic acid, methyl ester; linoleic acid ethyl ester; 3,4-dihydroxymandelic acid; and 13-docosenamide, (Z)- are reported to have an anti-inflammatory effect, which could account for the significant anti-inflammatory activity (Gomathi Rajashyamala and Elango, 2015; Kalinowska et al., 2018; Ley et al., 2002; Santos et al., 2013; Shareef et al., 2016; Sudha et al., 2013). There was a significant correlation between the anti-inflammatory activity seen in the *in vitro* assays and the *in silico* molecular docking simulations of the bioactive compounds. 3,4-Dihydroxymandelic acid showed the strongest docking interaction (-9.079 kcal/mol), followed by 2,5-dihydroxybenzoic acid (-8.996 kcal/mol), linoleic acid ethyl ester (-7.014 kcal/mol). The other compounds exhibited moderate docking scores.

A number of non-animal sources such as fruits and vegetables have been exploited as supplements having anticoagulant, antiplatelet, and fibrinolytic potential. Studies have shown that consuming such foods helps to prevent the occurrence of coronary incidents and stroke (Rahman et al., 2013). In our thrombolytic assay, a concentration of 10 mg/mL showed the maximum clot lysis activity (35.72%, *P* < 0.001) in relation to the negative control (normal saline; 4.49%), while streptokinase exhibited a 74.52% activity. Thus, thrombolytic activity could arise owing to the presence of 3-methylsalicylic acid; 11,14-eicosadienoic acid, methyl ester, and linoleic acid ethyl ester; these compounds reportedly have thrombolytic activity (Chen, 2019; Gomathi Rajashyamala and Elango, 2015; Sudha et al., 2013). The biologically active compounds and their thrombolytic activity were supported through the *in silico* molecular docking study. 3-Methylsalicylic acid showed the strongest docking interaction (-6.292 kcal/mol), which was stronger than that of the reference drug streptokinase (-6.173 kcal/mol).

An important prerequisite when assessing the anti-anxiety effect of a compound is that the drug should not elicit any motor deficit at the anxiolytic dosage, as this would be counterproductive to the desired test outcomes (Patel et al., 2013). OFT and the hole-cross test were used to assess locomotor activity, as well as the antidepressant effects, to evaluate the action of the drug in the central nervous system as a precondition test for anxiolytic action. The 200 and 400 mg/kg dosages of MECP did not elicit any motor deficit, which was evident by the locomotor activity testing. The decreased behavior that can be observed in locomotor testing has been utilized as a guide of CNS depressant action. The locomotor activity seen upon MECP administration might be related to the presence of 3,4-dihydroxymandelic acid, which reportedly has noradrenaline effects (Ley et al., 2002). The noradrenaline effects of 3,4-dihydroxymandelic acid were supported by the *in silico* molecular docking simulation in which it showed a lower docking score (-5.804 kcal/mol) with a strong favorable bond.

The elevated plus maze test, hole-board test, and light–dark tests were used to evaluate the anxiolytic activity in an unlearned or unpunished method. The elevated plus maze test is considered a better apparatus for the evaluation of anxiety behavior. In this test, increased entries and time spent in the open arms are considered to show an anxiolytic effect (M. Uddin et al., 2018). MECP elicited significant anxiolytic activity indicated by the increased time and entries in the open arms. A similar activity is assessed in the hole-board test, where head-dipping behavior is considered as indication for an anxiolytic response (Doukkali et al., 2015). In our study, the administration of MECP and diazepam elicited an extremely significant amount of head-dipping and also the latency for the first head-dipping compared to the control group. In the light–dark test, Swiss albino mice consistently attempted to spend time in the dark compartment due to the fear behavior when presented with a new condition. Transitions between the light and dark areas are considered as anxiolytic activity exploration (Bourin and Hascoët, 2003). MECP elicited a significant number of transitions to the light compartment compared to the control group, but it also decreased the time spent in the light compartment. The significant anxiolytic activity might be due to the presence of epinephrine (beta) in MECP, as well as the noradrenaline effects of 3,4-dihydroxymandelic acid (Ley et al., 2002). The noradrenaline effects of 3,4-dihydroxymandelic acid were supported by the *in silico* molecular docking simulation, where it showed a low docking score (-5.104 kcal/mol), which was higher than the reference drug diazepam (-3.035 kcal/mol) (Joel et al., 2004).

The antinociception or analgesic effect was evaluated by injecting acetic acid intraperitoneally and assessing the writhing response of mice. Both central and peripheral analgesia can be confirmed by applying the acetic acid test (Bellah et al., 2017). Pain is stimulated in the body due to the presence of several inflammatory mediators, e.g., histamine, prostaglandin, and bradykinin. In this experiment, pain perception was caused by inflammation in localized areas due to the release of free arachidonic acid via COX and prostaglandin synthesis (Ricciotti and FitzGerald, 2011). In the writhing test, the level of prostaglandin (prostaglandin E₂ and prostaglandin F2α) and lipoxygenase products increased markedly in the peritoneum cavity. NSAIDs are widely used due to their significant anti-inflammatory action at peripheral sites by blocking meditators (Sen et al., 2018). In our study, MECP elicited an extremely significant inhibition in writhing in a dose-dependent manner. The 400 mg/kg dose exhibited an inhibitory action similar to that of the standard drug diclofenac sodium. This action might be due to the presence of phytol and 13-docosenamide, (Z)-, as well as 3-methylsalicylic acid (salicylic acid derivative); 2,5-dihydroxybenzoic acid; and 3,4-dihydroxymandelic acid (Kalinowska et al., 2018; Ley et al., 2002; Santos et al., 2013; Shareef et al., 2016). The antinociceptive and anti-inflammatory effects were supported by *in silico* molecular docking. For the COX1 receptor, 3-methylsalicylic acid exhibited the lowest docking score (-6.982 kcal/mol), followed by 3,4-dihydroxymandelic acid (-6.29 kcal/mol) and 2,5-dihydroxybenzoic acid (-6.167 kcal/mol). For COX2, 2,5-dihydroxybenzoic acid (-6.721 kcal/mol) exhibited the strongest binding affinity, followed by 3,4-dihydroxymandelic acid (-6.413 kcal/mol) and 3-methylsalicylic acid (-6.111 kcal/mol).

In the antidiarrheal test, castor-oil is used as a laxative to induce an alteration in the water and electrolyte absorptivity in the intestinal mucosal layers, resulting in liquid and watery luminal contents that are rapidly eliminated by the intestines (Rizzo et al., 2016). Ricinoleic acid is a natural laxative and is the active metabolite of castor-oil. Castor oil acts on the small intestine to change the action of the smooth muscle GI (Ansari et al., 2017). In our study, castor oil-induced diarrhea was significantly inhibited at both doses, but the gastrointestinal motility test showed a lower amount of intestinal motility inhibition. These results might have been due to the presence of sorbitol, which is a laxative (Liauw and Saibil, 2019). The laxative effect of MECP was suggested by the *in silico* molecular docking simulation, where sorbitol had a score of -3.948 kcal/mol, while loperamide had a score of -8.152 kcal/mol.

Virtual screening is an important tool that enables researchers to assess the theoretical ADME/T profiles of compounds that have not yet been measured for their activity against specific drug sites (Ntie-Kang et al., 2013). ADME evaluation can be used to more accurately identify potentially active compounds based on their predetermined activity compared to random screening. In our ADME study, all of the selected compounds followed Lipinski's rule of five, which should be taken into account when identifying prospective drug targets.

## 5 Conclusions

Based on the present study, the methanol extract of *C. pectinata* is a potential source of phytochemicals that have significant anxiolytic and analgesic properties and strong antioxidant activity. In addition, a significant anti-inflammatory and blood clot lytic activity was observed. The GC-MS analysis helped to identify the presence of several bioactive compounds. Further advanced *in vivo* and *in vitro* studies are required to evaluate and isolate the pure compounds that are accountable for the biological effects.

## Declarations

### Author contribution statement

T.B. Emran: Conceived and designed the experiments; Analyzed and interpreted the data; Wrote the paper.

A.M. Tareq: Conceived and designed the experiments; Performed the experiments; Analyzed and interpreted the data; Wrote the paper.

S. Farhad, M. Hoque, Mir Md. Rokib Uddin, M. Hasan, A. Sultana, A.B.M. Neshar Uddin and Mst. Shirajum Munira: Performed the experiments.

Mst. Samima Nasrin: Contributed reagents, materials, analysis tools or data; Wrote the paper.

C. Lyzu: Performed the experiments; Contributed reagents, materials, analysis tools or data.

S.M.M. Hossen: Analyzed and interpreted the data; Contributed reagents, materials, analysis tools or data.

A.S.M.A. Reza: Conceived and designed the experiments; Analyzed and interpreted the data; Contributed reagents, materials, analysis tools or data; Wrote the paper.

### Funding statement

This research did not receive any specific grant from funding agencies in the public, commercial, or not-for-profit sectors.

### Competing interest statement

The authors declare no conflict of interest.

### Additional information

No additional information is available for this paper.

## Appendix A Supplementary data

Supplemenatry File Final Supplemenatry File Final
