# Supplementary material for: Chemical profiles, pharmacological properties, and in silico studies provide new insights on Cycas pectinata
Source: Heliyon. 2020 Jun 4;6(6):e04061. doi: 10.1016/j.heliyon.2020.e04061 (PMC7283161; doi:10.1016/j.heliyon.2020.e04061)
Supplement: Supplemenatry File Final [file mmc1.docx]

**Supplementary materials**

Table

Table 1S: Qualitative phytochemical screening of *C. pectinata* leaves.

| Test Name | Results |
| --- | --- |
| Alkaloid | + |
| Carbohydrate | + |
| Flavonoid | + |
| Terpenoids | - |
| Tannins | - |
| Saponins | + |
| Phenols | - |
| Polyphenol | - |
| Steroids | + |
| Cardiac glycosides | + |
| Glycosides | + |
| Coumarin | + |
| Amino acids | + |
| Resins | + |
| Quinones | - |
| Anthraquinones | - |
| Vitamin – C | - |
| Gums and mucilage | + |
| Carboxylic acids | - |
| Phytosterols | + |
| Sterols | + |

- = absent, + = present

**Figure**


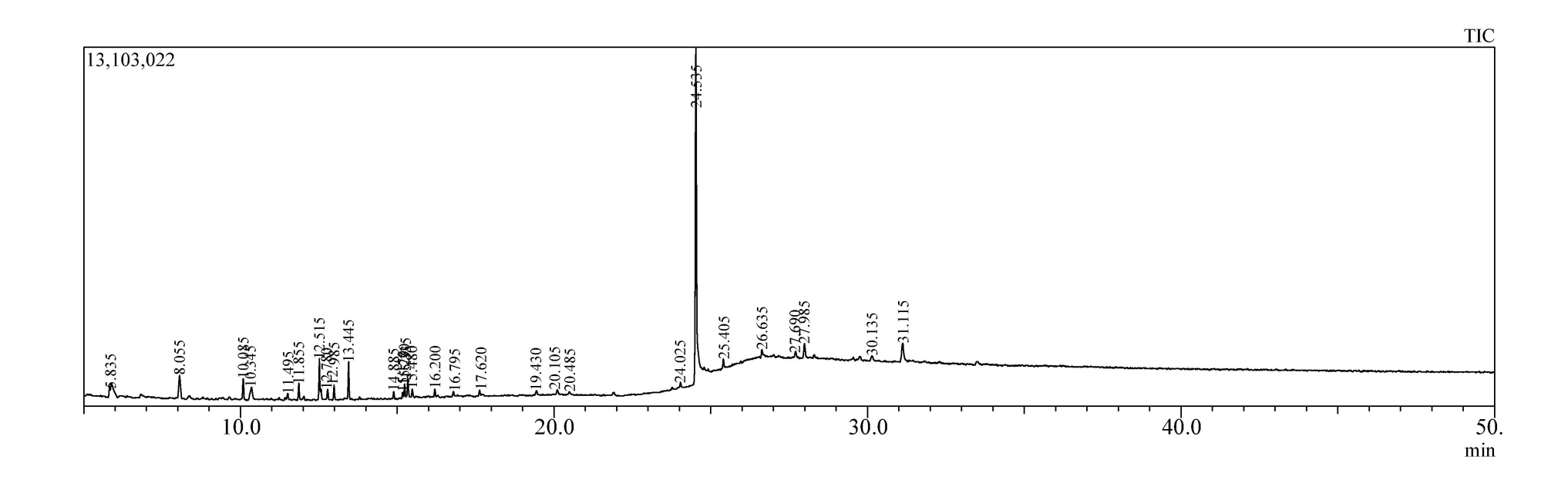


Figure 1S: Total ionic chromatogram (TIC) of methanol extract of *C. pectinata* leaves by GC-MS.
